# From maternal breath to infant's cells: Impact of maternal respiratory infections on infants ‘immune responses

**DOI:** 10.3389/fped.2022.1046100

**Published:** 2022-11-07

**Authors:** Nicolas Dauby, Véronique Flamand

**Affiliations:** ^1^ Institute for Medical Immunology, ULB Center for Research in Immunology, Université Libre de Bruxelles (ULB), Brussels, Belgium; ^2^ Department of Infectious Diseases, CHU Saint-Pierre, Brussels, Belgium; ^3^ School of Public Health, Université Libre de Bruxelles (ULB), Brussels, Belgium

**Keywords:** COVID-19, SARS-CoV-2, influenza, pregnancy, fetal immunity, microbiota, placenta, inflammation

## Abstract

*In utero* exposure to maternally-derived antigens following chronic infection is associated with modulation of infants ‘immune response, differential susceptibility to post-natal infections and immune response toward vaccines. The maternal environment, both internal (microbiota) and external (exposure to environmental microbes) also modulates infant's immune response but also the clinical phenotype after birth. Vertical transmission of ubiquitous respiratory pathogens such as influenza and COVID-19 is uncommon. Evidence suggest that in utero exposure to maternal influenza and SARS-CoV-2 infections may have a significant impact on the developing immune system with activation of both innate and adaptive responses, possibly related to placental inflammation. Here in, we review how maternal respiratory infections, associated with airway, systemic and placental inflammation but also changes in maternal microbiota might impact infant's immune responses after birth. The clinical impact of immune modifications observed following maternal respiratory infections remains unexplored. Given the high frequencies of respiratory infections during pregnancy (COVID-19, influenza but also RSV and HMPV), the impact on global child health could be important.

## Introduction

Early immune responses in humans, both during the immediate post-natal period and infancy are shaped *in utero* and modulated by both maternal and environmental factors (1). As such, exposure to chronic maternal infections, in the absence of vertical transmission, is associated with induction of innate immune responses, pathogen-specific adaptive immune responses (sensitization) and decreased transfer rate of maternal antibodies. Those immune abnormalities are linked to modified susceptibility toward homologous pathogens (i.e., pathogen to which the infant was exposed *in utero*) or heterologous infections and modifications of immune response toward vaccine at birth or during infancy (2–7).

*In utero* exposure to non-pathogenic microbial environmental factors have also an impact on the clinical phenotype during infancy. This include differential susceptibility toward occurrence of immune mediated diseases such as atopic dermatitis or asthma (2, 8).

Influenza infection and influenza-like illness are relatively common during pregnancy worldwide, in both low & middle and high income countries (9–11). For example, incidence rates ranged from 0.7 to 0.9% per month of pregnancy in a large multi-centric studies in 3 middle income countries (10). In the pre-COVID-19 vaccine era, up to 10% of pregnant or recently pregnant women were diagnosed with COVID-19 at hospital admission for any reason (12).

Both maternal influenza and COVID-19 infections are associated with increased maternal morbidity and mortality but also increased risk of adverse neonatal outcomes such as low birthweight and premature birth (12–16). Vertical transmission with COVID-19 or influenza is uncommon (16).

Respiratory infections, such as influenza and COVID-19, are associated with lung, systemic and also placental inflammation that could impact post-natal immune responses. Infections-associated modulation of the maternal microbiota could also have an impact on neonates' microbiota and infants ‘immune responses.

The aim of this mini review is to summarize the possible mechanisms and current knowledge linking maternal respiratory infections with modulation of neonatal immunity and immune-related health outcomes.

## The maternal respiratory environment's impact on fetal immunity: lessons from the allergy field

Different studies across the world have demonstrated that the respiratory environment and specifically the microbial respiratory environment of the mother can impact fetal immunity and also the clinical phenotype during infancy. T-cells sensitization toward allergens found in maternal homes have been detected in cord blood (17). Exposure to a farming environment, contaminated by mold and bacteria species, during pregnancy has been associated with differential expression of Toll-like receptors (TLR) genes at birth (18). Children born to mothers living in farm environments have decreased risk of asthma and atopic dermatitis during childhood as compared to children born to mothers not living in such environment (19).

The factors associated with farm exposure and modulation susceptibility to atopic disease is likely multifactorial (19). Animal models suggest that microbial exposure through the maternal respiratory tract likely contribute to both immune and clinical risk modifications. In a mice model, maternal intra-nasal exposure during pregnancy to a nonpathogenic bacterium (*Acinetobacter lwoffii*) was protective against the development of experimental asthma in the progeny (20). This protection was related to modulation of both maternal and placental immune responses. Maternal exposure to *A. lwoffii* was associated with increased levels of maternal lung pro-inflammatory cytokines (TNF, IL-6 and IL-12p40) and TLR mRNA (TLR-2, 3, 6, 7 and 9). On the other hand, a decreased expression of placental TLR mRNA (TLR 6 and 7) was observed along with suppression of pro-inflammatory placental cytokines production (IL-1 and TNF). Different knock-out experiments using TLR deficient mice suggest a critical role of maternal TLR signaling in the protection against asthma in their progeny.

Overall these data indicate that prenatal exposure to a specific maternal microbial respiratory environment is associated with modification of immune responses at birth with a potential clinical impact during infancy.

## Modifications of the maternal microbiota: impact on fetal immunity

Longitudinal cohort studies suggest that infant upper respiratory tract flora are acquired at least in part from their mothers (21). It has been suggested that the placenta is home to beneficial commensals (22) and that microbiota inhabit the fetal lung. Moreover, changes in microbiota composition during pregnancy influence fetal growth and development (23). Nowadays, the microbiome, defined as commensal living microorganisms, their genome, environment and the host interactions, is known to be responsible for the maintenance of homeostasis and tissue stability of different organs including the lung. The microbiome also impacts crucial event such as conventional dendritic cell maturation that will shape innate and adaptive immunity in early life (24). There is evidence that editing of the maternal gut microbiome with some beneficial bacterial strains during pregnancy may attenuate the features of allergic airways disease in the offspring through the stimulation of bone marrow hematopoiesis of dendritic cells (DC) precursors (25). A recent murine study demonstrates the role of maternal gut microbiome in regulating immunity to respiratory syncytial virus (RSV) infection in offspring. A prenatal *Lactobacillus* exposure through maternal supplementation led to decreased Th2 cytokines and lung inflammation following RSV infection in their neonates (26). Therefore, the interplay between maternal microbiota and the infant immunity may occur through microbiota editing or dysbiosis.

## In utero sensitization following maternal respiratory infections

During chronic maternal infections with malaria or helminths, *in utero* sensitization occurs and is associated with modification of infant's susceptibility toward homologous infection during infancy (27, 28)*.* As an example, placental malaria modifies the risk of malaria during infancy, possibly by the induction of a tolerant phenotype in exposed-infants (27).

*In utero* sensitization following maternal respiratory infection could possibly modulate the risk of infection in the post-natal period. Whether or not *in utero* sensitization to influenza occurs remains controversial: some historical studies have detected the presence of influenza-specific IgM (29, 30) in cord blood of exposed-newborns while a more recent study has not (11). Following maternal COVID-19 infection, no sign of SARS-CoV-2 sensitization has been reported by different studies, assessed by T-cells specific activation of IgM production in cord blood (31–33).

These observations indicate that *in utero* sensitization toward respiratory infections is uncommon following acute maternal respiratory infections, on the contrary to what is reported during maternal chronic infection or exposure to environmental antigens (2, 17).

## Maternal respiratory infections and placental inflammation

During maternal infection, placental inflammation could occur following direct placental infection or systemic inflammation.

Since the beginning of the COVID-19 pandemic, various studies by different groups have assessed the impact of maternal COVID-19 infection on placental inflammatory responses by directly studying placenta from SARS-CoV-2 infected mothers or by using models of placental explants models (32, 34–37).

*Ex vivo* models of placental infection indicate that SARS-CoV-2 is able to replicate and propagates in human placenta (37). Placental SARS-CoV-2 RNA is detected at high frequency following late maternal infection during pregnancy. Subgenomic RNA was found in 22/52 (42%) of a cohort of mothers who were tested positive at delivery and was localized to the trophoblasts (35). However, no live virus was detected. Important immune cells infiltration is found in the placenta of infected mothers (34). SARS-CoV-2 infected placenta explants display strong inflammatory responses with increased expression of chemokines, inflammations pathways and apoptosis/necrosis related genes (35).

The impact of maternal influenza infection during pregnancy on placental pathology and inflammatory responses has been assessed in mice models. Following maternal influenza infection, no viral RNA is detected in the placenta or in the fetal tissue, in line with the absence of vertical transmission documented in humans. However, strong IFN responses are detected in the placenta (38) along with production of inflammatory chemokines (RANTES and G-CSF) (39).

In summary, these observations from both clinical and animal studies, indicate that maternal respiratory infections during pregnancy, in the absence of vertical transmission, induce strong inflammatory responses in the placenta with production of chemokines, leading to immune cell infiltrations.

## Maternal respiratory infections' impact on fetal immune responses

Since the start of the COVID-19 pandemic, different human studies have been performed on the impact of maternal SARS-CoV-2 infection on neonatal immunity shedding light on the potential impact of a respiratory infection on neonatal immunity.

An important study that included three groups of mothers: non-SARS-CoV-2 infected, recovered infection and one with recent/ongoing infection found a gradient in the magnitude of the innate immune responses induced in cord blood. Those inflammatory responses included expansion of innate immune cells such as *γδ* T cells and NK cells and production of pro-inflammatory chemokines (CXCL8). Fetal immune cells show signs of activation as assessed by TNF-α and IFN-*γ* production. Immune activation is probably non-specific as no sign of SARS-CoV-2 sensitization, has been observed (31).

In a similar study that included 11 PCR-confirmed, mostly asymptomatic mothers, higher IL-8 production was found in cord blood of SARS-CoV-2-exposed neonates. Interestingly, exposure to SARS-CoV-2 *in utero* was associated with differential expression of genes involved in defense toward bacteria and fungus (32). Mild decrease of reactive oxygen species production by monocytes of SARS-CoV-2 exposed neonates was also reported.

Altogether, these data suggest that exposure to SARS-CoV-2 during pregnancy is associated with significant fetal immune activation, independent of vertical transmission and is probably related to placental inflammation more than transfer of viral byproducts. Importantly, those immune modifications have been reported in neonate born to asymptomatic mothers, suggesting that the long term impact could involve large numbers of neonates.

No human studies have however explored the impact of maternal influenza infection on fetal immunity; although different mice models provided insight on the potential impact of maternal influenza infection on fetal immunity. In one study, a downregulation of genes associated with Toll-like receptor signaling and T cell differentiation in fetuses was observed, suggesting a potential impact on infants post-natal immune responses (40). In another study that also used a model of maternal intra nasal influenza A virus (IAV) infection, *in utero* exposure to influenza was associated with reduced frequencies of NK and B cells in the lungs from offspring born to IAV-infected dams. Adoptive transfer experiments of alveolar macrophages from offspring born to uninfected-dams to offspring born to IAV-infected dams suggested an impairment of the function of alveolar macrophages secondary to *in utero* influenza infection. Indeed, offspring exposed to influenza *in utero* showed better recovery following influenza B virus infection after transfer of alveolar macrophages from offspring born to IAV-uninfected dams (41). Finally, elevated levels of the pro-inflammatory cytokine IL-1 was found in the cord blood of offspring born to influenza-infected dams (39).

In summary, evidences from both clinical and animal studies, indicate that exposure to respiratory infections during pregnancy, including mild disease, is associated with expansions and activation of innate immune cells, with a possible impact on the functionality of innate cells such as monocytes and lung macrophages.

## Clinical impact of the exposure to maternal respiratory infections

The profound clinical impact during childhood of *in utero* exposure to maternal influenza infection has been reported since the 1,918 influenza pandemic (14, 42). Immediate post-natal health impact of maternal influenza infection include stillbirth, low birth weight and prematurity. Congenital heart defects and neurological and behavioral changes such as schizophrenia are also reported during childhood after maternal influenza infection. Large nationwide studies have also shown long-term effect of maternal influenza infection with increased morbidity during adulthood (43–45). Preliminary data suggest that *in utero* exposure to SARS-CoV-2 during pregnancy might impact neurodevelopmental outcomes at 1 year of age (46).

The impact of both maternal influenza and SARS-CoV-2 infection on the susceptibility to infections during infancy has not been assessed in humans. In a mice model of maternal influenza A infection, exposed neonates were more susceptible to heterologous infections challenges (influenza B and MRSA), possibly related to alveolar macrophages dysfunction (41). Studies are currently underway to assess the long-term health consequences of *in utero* SARS-CoV-2 exposure, including infectious risk during infancy.

## Discussion and perspectives

We have reviewed the different mechanisms associated with acute maternal respiratory infections that could possibly impact infant's immune responses.

Previous research in the field of allergy and chronic maternal infections have highlighted the impact of maternal exposure to specific microbial respiratory environment and immune modulation related to chronic infections on infant's immune responses. Susceptibility toward infection and response to vaccines in infancy are modulated after exposure to chronic maternal infection, without vertical transmission while susceptibility toward immune-mediated diseases (asthma, atopic dermatitis) is impacted by the respiratory maternal environment (2, 8, 19).

In this mini-review, we show that part of these mechanisms are also observed during maternal respiratory infections, namely induction of innate immune responses and placental inflammation. On the other hand, *in utero* sensitization seems infrequent after exposure to maternal respiratory infections such as influenza and COVID-19.

Maternal microbiota editing may also have an impact on infants ‘immune responses. During pregnancy, disturbance in the mother microbiota either as a result of infection or stress linked to sexual hormones might influence infant health through several ways. Following lung-gut microbiota crosstalk, additional effects may be associated with upper respiratory infections during pregnancy. Alterations in the lung microbiota profile have been associated with several respiratory diseases such as pneumonia or viral infection (47). Dysbiosis of the lung and gut microbiomes have been also observed in severe COVID-19 patients (48, 49) and following influenza infection in mice and humans (50, 51). How prenatal events such as mother's infection influence the induction and training of a healthy maturation of the innate and adaptive immunity in early life still need further investigations. How the materno-fetal microbiota alterations associated with respiratory infections may affect the ontogeny of neonatal innate and adaptive immunity is underexplored and warrants further research.

Maternal respiratory infections such as influenza and COVID-19 are associated, beside upper and respiratory tract inflammatory responses, with systemic and placental inflammatory responses. Studies performed during the COVID-19 pandemic indicate that neonates exposed to respiratory infections have distinct innate but also adaptive responses, independently of virus-specific immune activation, as sensitization appears infrequent (31–33). Results from both humans and animal studies suggest that anti-microbial immune responses might be affected following exposure to viral respiratory infections during pregnancy (32, 40, 41). Differential expression of genes involved in anti-microbial responses (32) are reported following COVID-19 infection. Altered functionality of monocytes and alveolar macrophages are described following maternal respiratory infection, which might impact susceptibility toward post-natal bacterial infection, as shown in a mice model (41). Previous research in the field of chronic maternal HIV infection has highlighted the role of neonatal immune activation at birth on infectious risk during infancy. Indeed, HIV-exposed but uninfected infants born to mothers who initiated anti-retroviral therapy during pregnancy have higher risk of hospitalization for infection as compared to HIV-unexposed infants and this risk is related to activation of monocytes at birth (6). These observations warrant clinical studies assessing the risk of infections in early life following respiratory infections.

Modulation of placental TLR responses is associated with differential risk of atopic diseases during infancy. The impact of maternal influenza or COVID-19-induced placental inflammation on the risk of atopic diseases during infancy is undetermined.

Finally, responses to vaccines at birth or during infancy, might also be impacted following disturbances of both adaptive and innate immune responses, as previously shown following chronic maternal infections with protozoans or helminths (3, 7). Notably, decreased transfer of vaccine and pathogens-specific IgG are observed following chronic maternal infections (2). The mechanisms involved possibly include hypergammaglobulinemia (52) but also placental inflammation, with subsequent alteration of IgG transfer through the Fc neonatal receptor (2). Hypergammaglobulinemia has not been reported during acute maternal COVID-19 or influenza infection but placental inflammation could impact the transfer of maternal IgG. Given the increasing role of maternal immunization in the prevention of maternal and neonatal morbidity (53), more studies are required to assess the impact of both influenza and COVID-19 on the transfer of maternally-derived IgG specific for current and upcoming vaccines such as pertussis, tetanus, Group B streptococcus and RSV.

Most of the literature reviewed here was related to influenza and COVID-19. Other respiratory infections such as RSV and human metapneumovirus (HMPV) are highly prevalent worldwide and also reported during pregnancy as causes of acute respiratory illnesses (54–56). More research is needed to assess the impact of maternal RSV or HMPV infections on placental and infants ‘immune responses.

Maternal vaccination is recommended during pregnancy for both influenza and COVID-19; whether maternal vaccination is associated with dampening of inflammatory immune response following respiratory infection remain to be demonstrated.
